# Targeting histamine H4 receptor improves anti-tumoral response in a murine model of breast cancer

**DOI:** 10.3389/fimmu.2026.1770957

**Published:** 2026-04-21

**Authors:** Ramiro Vázquez, Paula Saibene, Maria Emilia Boix, Mariano Maio, Gabriela Salamone, Luciana Balboa, Mónica Vermeulen

**Affiliations:** 1 Laboratorio de CPA y respuesta inflamatoria, Instituto de Medicina Experimental (IMEX)-Conicet, Academia Nacional de Medicina, Pacheco de Melo 3081 (CP1425AU), Buenos Aires, Argentina; 2 Instituto de Investigaciones Biomédicas en Retrovirus y SIDA (INBIRS) Facultad de Medicina, Universidad de Buenos Aires - Consejo Nacional de Investigaciones Científicas y Técnicas (CONICET), Buenos Aires, Argentina; 3 Departamento de Inmunología, Facultad de Medicina, Universidad de Buenos Aires, Buenos Aires, Argentina

**Keywords:** 4T1 murine breast carcinoma, CD8 lymphocytes +, ERK1/2, histamine H4 receptor, metabolism

## Abstract

**Background:**

Breast cancer is one of the most common malignant tumors in women worldwide. Histamine (HIS) has been associated with either pro-tumor or anti-tumor effects, depending on the receptor evaluated. It exerts its physiological and pharmacological functions through four receptors (H1R-H4R). In breast cancer, it promotes tumor growth by activating H1 and H2 receptors. Furthermore, HIS released into the tumor microenvironment can promote inflammation associated with tumor development and immunosuppression of the immune response. Several years ago, breast cancer was shown to express the H4R, although conflicting data exist; its activation is associated with tumor progression and the development of metastases.

**Methods and Results:**

In this study, using a murine model of breast cancer with the 4T1 cell line, we investigated in depth the role of H4R in tumor progression. We demonstrated that H4R blockade with the specific antagonist JNJ777120 (JNJ) inhibits tumor cell line proliferation, migratory capacity, and ROS production, while increasing lactate release and rapidly and transiently ERK kinase activation. *In vivo*, the antitumorigenic effect of JNJ appears to depend on the adaptive immune response, as suggested by the rapid recruitment of CD8^+^ cells, the increased lymphocyte proliferation from JNJ-treated tumors, and the absence of activity in the adaptive immune-deficient Rag1 mice. Moreover, tumor-derived cells showed altered energy metabolism.

**Conclusion:**

Taken together, our results demonstrate the dual role of HIS via the H4R in 4T1 cells, modulating not only tumor cell development but also the immune microenvironment.

## Introduction

Breast cancer (BC) is the most common malignancy in terms of incidence worldwide (https://www.who.int/news-room/fact-sheets/detail/cancer). Multiple actions are required to reduce mortality, particularly in tumors with aggressive behavior, such as the triple-negative breast cancer (TNBC), lacking the so well exploited targets in forms: estrogen and progesterone receptors and the human epidermal growth factor receptor 2 (HER2) (1).

Histamine (HIS) is a biogenic amine with a central role in inflammation and allergy. Its functions are associated with four receptors (H1R, H2R, H3R, and H4R) (2). The H4R was described in leukocytes, and its expression level determines their pro or anti-inflammatory functions. Interestingly, histidine decarboxylase (HDC), the responsible enzyme of HIS production, is expressed in several tumors, comprising melanoma, colon and BCs. Indeed, certain polymorphisms of the HDC gene have been reported to correlate with BC (3, 4). In addition, data analyses in BC patients have revealed decreased HDC (mRNA) expression in malignant cells compared to adjacent normal tissue. Even more, samples from the most aggressive subtypes, such as TNBC, presented the lowest *hdc* transcription. In line with this, increased HDC mRNA levels statistically correlated with improved disease-free survival in all BC patients, including TNBC cases, suggesting that HIS may exert a metastasis-delaying effect in BC (5). Tellingly, similar conclusions were drawn when H4R expression was analogously analyzed (6, 7).

Despite the above, the application of HDC and/or H4R as prognostic markers requires much more research. That becomes evident by considering the vast contradictory literature regarding the character of HDC and/or HIS in cancer progression. Nevertheless, rather than focusing on their role as pro- or anti-neoplastic, more emphasis should be placed on the context, taking into account the type of tumor and its microenvironment (TME) features, like the HIS concentration and, specially, the receptor/s involved.

In this sense, the immune system also plays a key role in the TME detecting and eliminating cancer cells, so avoiding the development and propagation of malignant tumors. If this immune surveillance is not competent to counteract tumor growth, the resulting clinical frame will require therapeutic approach. Even in these circumstances, a competent immune system is crucial for the chemo-, immuno- and/or radiotherapy to be successful (8–10). In an *in vivo* model of BC orthotopically inoculating syngeneic tumor-derived 4T1 cells in BALB/C mice, Nicoud MB et al. showed that, compared to controls, neoplastic tissue from animals daily treated with subcutaneous administration of HIS (5 mg/kg) presented a substantial reduction in the mitotic index and increased number of apoptotic cells, resulting in the shrinkage of tumors. Surprisingly, no changes in terms of leukocyte infiltration accompanied such effect (5). This lack of immune surveillance would be a direct action of HIS on H4R as supported by the reproduction of the aforementioned experiment in H4R-knock out BALB/C animals (7, 11, 12) In this respect, HIS anti-tumor activity correlated with augmented CD4^+^ and CD8^+^ infiltrating lymphocytes in the malignant tissue.

Although the *in vitro* and *in vivo* tumor-inhibiting action of HIS has been already proved (5, 7), the mechanisms underlying both its direct effect on cancer cells as well as its modulatory influence on immune cells in the TME still remain intriguing. In particular, we are of the opinion that identifying the specific role each HIS receptor would not only contribute to the understanding of these aspects, but also raise new prognostic indicators and/or therapeutic targets.

Based on this, we aimed to characterize the role of H4R-mediated HIS signaling in the initiation of BC, the latter modelled by 4T1 cells both *in vitro* and in *in vivo*. In this framework, *in vitro* we found that the specific H4R blockade with the antagonist JNJ777120 –onwards JNJ– inhibited the cell proliferation and migration, which correlated with arrest in the G0/G1 cell cycle phase, altered oxidative metabolism, prompt and transient activation of ERK signaling pathway and changes in the expression pattern of membrane antigens. In the *in vivo* setting, a 30 min (10 M) JNJ treatment of the cells immediately before their inoculation was sufficient to inhibit the neoplastic growth. Moreover, increased recruitment of CD8^+^ lymphocytes and IFN-γ levels were detected in the TME one week after implantation of cells. In summary, our results not only support H4R as a primary factor in the development of BC, but also indicate that its blockade in cancer cells impacts the immune response developed in the TME.

## Materials and methods

### Mice

All experiments were conducted using 2-month-old virgin female BALB/c mice raised at the National Academy of Medicine, Buenos Aires, Argentina. They were housed six per cage and kept at 20 ± 2°C under an automatic 12-hour light-dark schedule. Animal care was under institutional guidelines. All experimental protocols were approved by the Institutional Animal Care and Use of the Experimentation Animals Committee (CICUAL number 125/2024).

### Cell culture and tissue disaggregation

All these experiments were carried out employing 4T1, lymph node- or tumor-isolated cells cultured in RPMI 1640 medium (Gibco) containing 10% fetal bovine serum (FBS, Natocor) and penicillin and streptomycin antibiotics (Sigma) and 5.5x10^5^ M β-mercaptoethanol (Sigma-Aldrich); complete medium (CM), being incubated at 37°C under a 5% CO_2_ atmosphere.

Lymph nodes and tumor tissues were removed, excised, placed in a Petri dish with RPMI 1640 medium, and mechanically dissociated by passing them through a sterile mesh to obtain a single-cell suspension of mononuclear cells. Then, cells were collected in conical tubes and centrifuged at 300-400 g for 10 min at 4°C. Finally, mononuclear cells were resuspended in CM for further subsequent analyses.

### Determination of cell growth by crystal violet staining

Exponentially growing 4T1 cells were seeded at a density of 3x10^3^ cells in 200 µl of RPMI 1640 containing 10 µM JNJ or 0.1% (v/v) DMSO (vehicle control group; Ct) in a 96-well culture plate, and incubated for 72 h. Cells were then washed with PBS and stained with 50 µl of 0.1% (s/v) crystal violet (7) in H_2_O at room temperature and mild agitation for at least 30 minutes. After that, wells were washed thrice with 200 µl water. Once the plate was dry, crystal violet was dissolved with 50 µl acetic acid 30% (v/v) in H_2_O, and absorbance was measured at 550 nm using an Asys UVM 340 microplate reader (Biochrom). Differences between samples were analyzed by employing Prism 5.00 for MS Windows software (Graph Pad Software) in at least three independent experiments carried out in triplicates.

### Proliferation assay

Mononuclear cells from lymph nodes and/or tumors were labeled with fluorescent dye CFSE (5 µM) and seeded in 96-well plates containing a monolayer of 4T1 line or anti-CD3 (0.1 µg/ml) at a concentration of 5x10^5^ cells/well. After 96 h, cells were recovered and CFSE fluorescence was evaluated by flow cytometry. Proliferating cells are those that present less intensity of fluorescence due to CFSE dilution in successive cell divisions.

### Cell cycle

After 72 h of incubation, Ct and JNJ-treated cells (10^6^ each) were detached with trypsin-EDTA solution, washed and resuspended in PBS. Subsequently, they were fixed and permeabilized by vigorous addition of three volumes of ice-cold 100% (v/v) ethanol and stored at −20°C for a minimum of 24 h, prior to analysis. Cells were resuspended in 800 µl of staining solution (20 µg/ml propidium iodide (PI) and 200 µg/ml RNase A in PBS) and incubated in the dark at 4°C overnight. The percentages of cells in the sub-G0/G1, G0/G1, S and G2/M cell cycle phases were determined by using FACS Calibur cytometer (Becton Dickinson) and data from at least three independent experiments were analyzed using Cyflogic free software (www.cyflogic.com).

### Wound healing assay

Cells were seeded at 5x10^5^ in 2 ml complete medium in 6-well plates and incubated for 24 h or until they reached > 90% confluence. At that time, cells were wounded by dragging a 1-ml pipette tip through the monolayer. They were washed with PBS to remove cellular debris and allowed to migrate for 48 h in RPMI 1640 medium with 1% FBS.

Wound closure or cell migration images were taken when the scrape wound was introduced (0 h) and every 24 h by employing a DMi6000 inverted microscope (Leica). The relative surface traveled by the leading edge was assessed using LAS AF6000 Version 1.8.0 software. The individual gaps were measured at each time point. Three independent experiments were carried out in triplicate.

### Flow cytometry

4T1 cells, and cells from tumors or lymph nodes (5x10^5^) were stained with monoclonal antibodies indicated in (**Supplementary Table S1**), conjugated with different fluorochromes for 20 min at 4°C. After incubation, cells were washed with cold PBS and resuspended in Isoflow™ (BD Pharmingen) until its acquisition. To perform intracellular staining cells were fixed in 0.5% paraformaldehyde and using the eBioscience™ FOXp3/Transcription Factor Staining Buffer set (cat. no. 00-5523-00, ThermoFisher Scientific). Permeabilized cells were incubated with PE-conjugated anti-mouse FOXp3 antibodies (BD Pharmingen) or isotype-matched control antibodies, for 30 minutes. Finally, cells were washed twice with permeabilization buffer, suspended in Isoflow, and analyzed by flow cytometry. In most cases, mononuclear cells were permeabilized to identify anti-CD25, anti-CD4 and anti-CD8 antibodies. Data were analyzed using a FACSCalibur™ flow cytometer (BD Pharmingen) or a Sysmex Partec Cyflow Space and were analyzed with Flowjo software.

### Degranulation assay

To determine the cytolytic activity of CD8 lymphocytes, we performed a degranulation assay. For this, 5x10^5^ mononuclear cells from tumors (14 days), treated or not with JNJ, were incubated on a 4T1 cell monolayer for 1 hour at 37°C. At the end of the incubation period, monensin (2 µM) was added to prevent granules from leaving the cell, along with Lamp1-FITC (CD107a), and the cells were incubated at 37°C for 4 hours. Finally, the cells were collected, thoroughly washed with cold PBS, and labeled with CD8 PE for 20 minutes at 4°C. Finally, the percentages of CD8^+^ Lamp1^+^ cells were determined by flow cytometry.

### Mitochondrial and oxidative metabolism

Cells (5x10^6^ cells) were resuspended in 1 ml of CM and treated with 10 µM JNJ or 0.1% DMSO (vehicle control) in 1.5 ml conical tubes for 30 min. Cells were then washed, and intracellular ROS and mitochondrial superoxide levels were assessed using the oxidative conversion of the cell-permeable probe 2′, 7′-dichlorofluorescein diacetate (DCFH-DA, 10 μM, Thermo Fisher Scientific) to fluorescent dichlorofluorescein (DCF), and the MitoSOX Red dye (2.5 μM, Thermo Fisher Scientific), respectively. Mitochondrial membrane potential was evaluated using the JC-1 probe (5 μM, Thermo Fisher Scientific). In all cases, the manufacturer’s instructions were followed. Briefly, after treatments, cells were washed with PBS, resuspended in FBS-free medium, and incubated with the aforementioned dyes for 15 min at 37°C. Dye-only controls were included in each experiment. Cells were then washed twice with PBS, and fluorescence intensity per cell was analyzed by flow cytometry using a FACSCalibur cytometer (Becton Dickinson).

### Determination of metabolites

Lactate production and glucose concentrations in the culture medium was determined employing the colorimetric assays Lactate Kit and Glicemia Enzimática AA Kit both from Wiener (Argentina), which are based on the oxidation of lactate or glucose, respectively, and the subsequent production of hydrogen peroxide (13). The consumption of glucose was determined by assessing the reduction in glucose levels in culture supernatants in comparison with RPMI 10% FBS. The absorbance was read using a Biochrom Asys UVM 340 Microplate Reader microplate reader and software.

### Cytokine determination

Cytokine levels in supernatants of mononuclear cells from lymph nodes or tumors, were measured by ELISA. Assays for IL-1β, tumor necrosis factor-(TNF-α), interferon-(IFN-γ), and IL-10 (eBiosciences) were performed according to the manufacturer’s protocols.

### Western blot analysis

4T1 cells were suspended in RPMI 10% fetal calf serum (2, 5-5x10^6^/ml) and treated or untreated with JNJ (10 μM) for different times at 37°C, finally washed with PBS. Pellets were resuspended in loading buffer (100 mM TRIS-HCL pH 6.8;4% SDS, 0.2% bromophenol blue, 20% glycerol and 200mM dithiothreitol), heated for 5 min at 95°C and frozen at -80°C. Proteins were separated onto 10% SDS-PAGE followed by electroblotting on membranes. These were blocked in PBS containing 5% milk for 1h and then incubated with the primary antibody in blocking buffer (PBS + 1% BSA + 0.05% Tween 20) overnight at 4°C: ERK1/2 (1:1000, Cell Signalling Technology). After washing secondary antibodies were applied in blocking buffer for 1 h at room temperature: anti- anti-rabbit (1:5000, Cell Signalling Technology). Specific bands were developed by chemiluminiscence (ECL, Amershan Biosciences). Membranes were reprobed with a rabbit mAb against murine β-actin (1:2000, Cell Signalling Technology). The quantification was performed using the ImageQuant program.

### Tumor growth study

Mice were subcutaneously injected in the right flank with 10^6^ 4T1 cells resuspended in PBS (control) or PBS + 10 µM JNJ. Neoplastic tissue growth was monitored every 72h by measuring their minor (*d*) and major (D) diameters. Tumor weight (1 mm^3^ = 1 mg) was determined using the formula *d*^2^ × D/2. Mice were sacrificed at the first sign of severe distress. On day 7 and 14 mice were euthanized by cervical dislocation and tumors and adjacent (subcutaneous) lymph nodes were collected processed as described above.

### Blood analysis

Heparinized blood samples (< 500 µl) were periodically collected by sub-mandibular bleeding using a lancet. Aliquots were diluted (1/20) in PBS and processed with an Abacus Junior Vet coulter (Diatron) to determine white cells populations following manufacturer’s instructions.

### Statistical analysis

Statistical analyses were performed using GraphPad Prism 6 (GraphPad Software). Graphs were generated using FlowJo X (FlowJo) and GraphPad Prism 6. Sample sizes (n) for each experiment are indicated in the corresponding figure legends and represent independent replicates unless otherwise specified.

For comparisons involving multiple groups, statistical significance was determined using one-way analysis of variance (ANOVA) followed by Tukey’s *post hoc* test. Comparisons between two groups were performed using Student’s t-test. A *p*-value < 0.05 was considered statistically significant.

## Results

### H4R blockade induces a cytostatic effect in the 4T1 cell line *in vitro*

**Figure 1:**
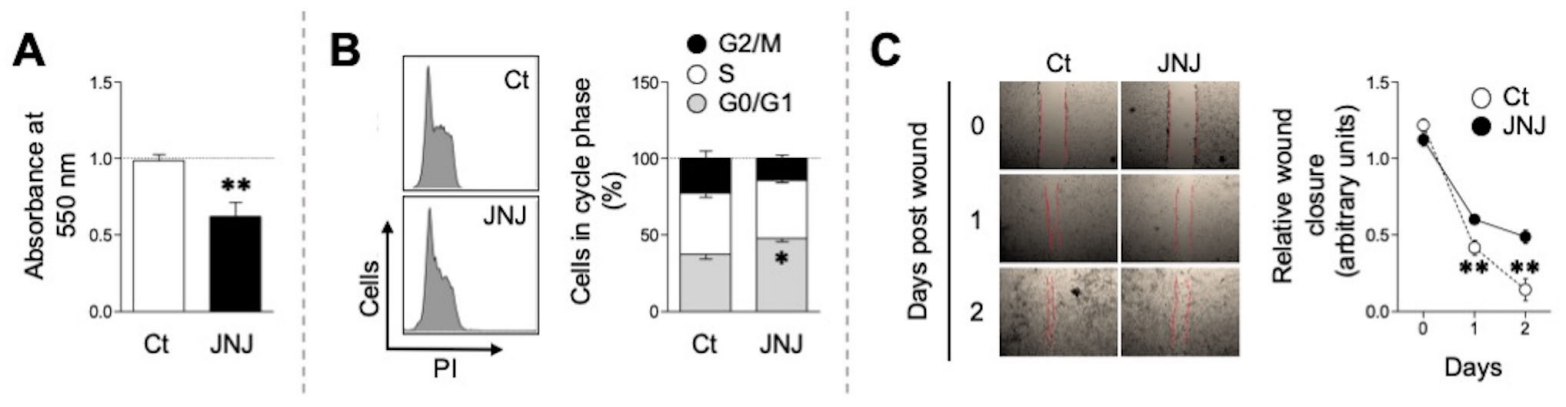
*In vitro* growth-inhibitory effect of JNJ. 4T1 cells were treated with 10 µM JNJ or 0.1% DMSO (Ct) for 72 h to evaluate proliferation [**(A)** n = 7] and cell cycle profile [**(B)** n = 4]. Each bar and vertical line represent the mean ± SEM, respectively. **(C)** The effect of JNJ on the migratory capacity of the cells was assessed by a wound-healing assay after a 24-h treatment. Scratch closure was monitored for 48 h following drug washout. Each dot and vertical line represent the mean ± SEM, respectively (n = 4). Significant differences in cell number **(A)**, in the percentage of JNJ-treated cells in the G0/G1, S, and G2/M phases **(B)**, and in wound closure at the indicated times **(C)**, were determined relative to Ct using Student’s t-test (*p < 0.05, **p < 0.01).

First, we assessed the *in vitro* capacity of JNJ (10 µM) to modulate the proliferation of the 4T1 cell line after 72 h of incubation. As shown in **Figure 1A**, this treatment significantly inhibited (*p* < 0.01) the cell growth compared to the vehicle (0.1% DMSO, onwards Ct), evaluated by optical density. This effect was associated with a clear arrest (*p* < 0.05) in the G_0_/G_1_ cell cycle phase (**Figure 1B**). Of note, no signs of cell death –such as cellular debris–, were observed in the cultures, nor were cells in the sub G_0_/G_1_ phase of the cycle (**Figure 1B**, left panel), supporting the cytostatic rather than cytotoxic effect of JNJ on the tumor cell line.

In line with the above, the wound healing assay showed a reduced proliferative and migratory capacity in those cells exposed to 10 µM JNJ *versu*s Ct (*p* < 0.01, **Figure 1C**). It is worth noting that, in this case, the treatments lasted 24 h and the cells were subsequently washed, giving us the first indication that the drug may have an early impact on the cell physiology with lasting outcomes.

### Evaluation of signaling pathways and phenotypic effects following H4R blockade

**Figure 2:**
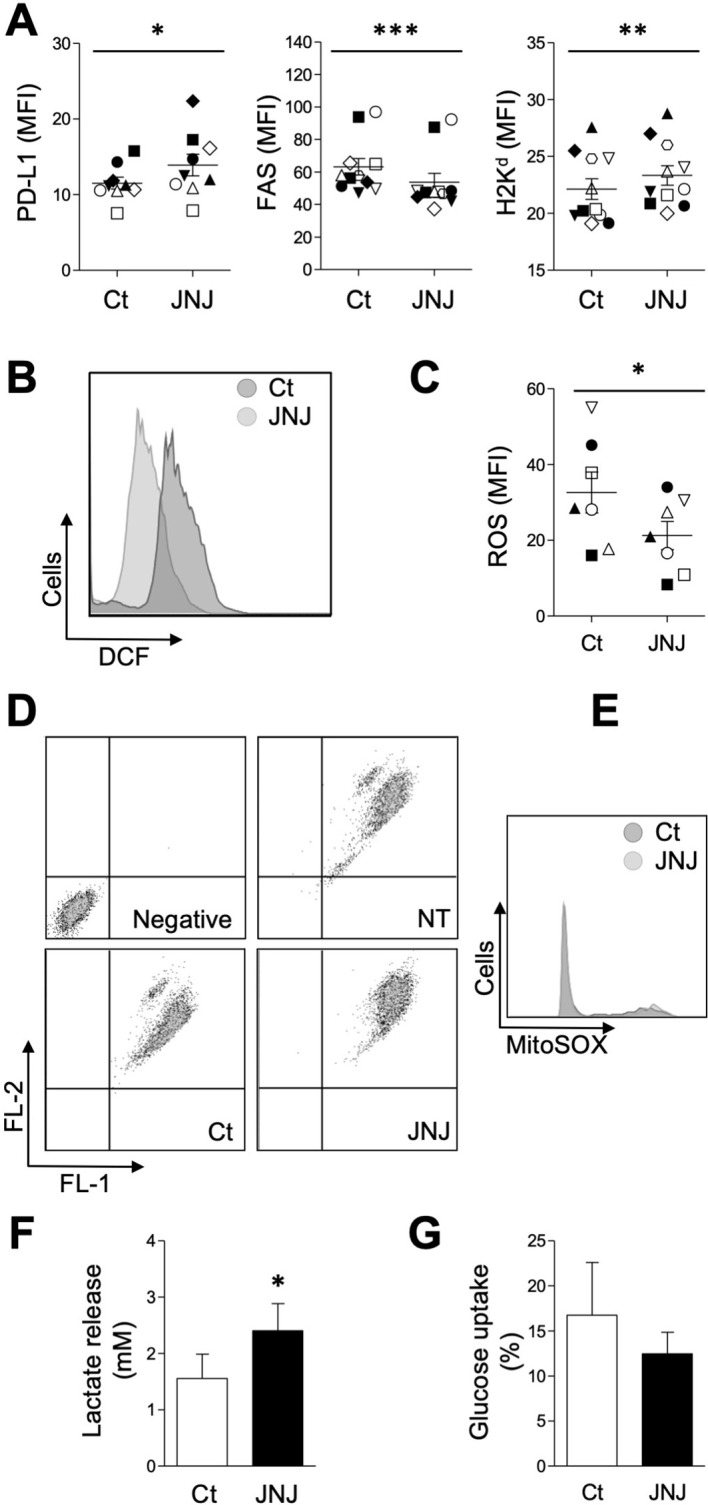
JNJ-induced alterations in 4T1 cells phenotype. **(A)** Cells were treated with 10 µM JNJ or 0.1% DMSO (Ct) to evaluate the membrane expression of PD-L1 (n = 9), FAS (n = 11), and H2Kκ (n = 11) by flow cytometry. ROS generation was assessed by H_2_DCFDA oxidation [**(B, C)** n = 7]. The fluorescence dot plot **(D)** indicative of mitochondrial membrane potential and the histogram of mitochondrial superoxide production **(E)** are representative of 4 experiments. Flow cytometry was performed after 30 min of treatment with JNJ or Ct. In **(D)**, the dye acts as a potential-dependent probe: it aggregates in healthy mitochondria with high potential (FL2 emission) and remains in monomeric form in depolarized mitochondria (FL1 emission). “Negative” refers to cells that were not treated (NT) and were not exposed to JC-1. Lactate secretion **(F)** and glucose uptake **(G)** were measured after a 24 h incubation with 10 µM JNJ or vehicle. In **(A, C, D)**, differences between treatments were determined using Student’s t-test (*p < 0.05, **p < 0.01, ***p < 0.001). In **(A, C)** dots and vertical lines represent mean fluorescence per cell ± SEM, respectively. Results from the same experiment are represented with identical symbols to illustrate overall consistency between replicates. In **(D, E)** bars and vertical lines represent mean ± SEM, respectively.

Next, we studied inhibitory and class I molecules associated with tumor scape (10, 14, 15) by cytometry. As shown in **Figure 2A**, the expression of PDL-1 and class I molecules increased after 24 h of culture in the presence of JNJ, while the expression of FAS was reduced. Taking into account, the relevance of energetic metabolism in cancer development (16, 17), we tested whether JNJ affected it. In this regard, the drug significantly reduced (*p* < 0.01) the production of reactive oxygen species (ROS), as assayed by H2DCFDA oxidation by flow cytometry, after a 30-min incubation (**Figures 2B, C**), suggesting either a direct antioxidant property of JNJ or an immediate effect on ROS-generating systems. It is worth mentioning that we found no effect on mitochondrial integrity (**Figure 2D**) or membrane potential (**Figure 2E**), measured in the presence of MitoSOX and JC-1 respectively, mediated by JNJ. To assess whether this rapid change was associated with longer-term metabolic reprogramming, we indirectly measured glycolytic activity by determining lactate release into medium and glucose uptake. **Figure 2F** shows that lactate levels significantly increased (*p* < 0.05) after a 24 h incubation with JNJ, whereas glucose consumption remained unchanged (**Figure 2G**). Notably, although JNJ ultimately inhibits proliferation, it stimulates lactate production, which is frequently associated with tumor aggressiveness (18, 19). As we showed, the metabolic changes did not involve either loss of mitochondrial integrity or alteration of the membrane potential; we believe that the dysfunction would involve activation of non-mitochondrial mechanisms such as the regulation of NADPH oxidase or NADH^+^-dependent redox modulation.

**Figure 3:**
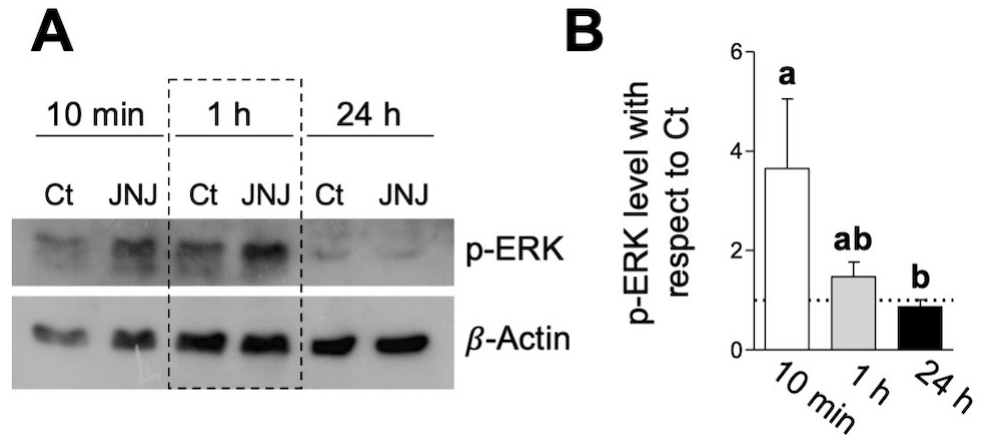
JNJ modulation of the ERK signaling pathway. **(A)** Cells were treated with 10 µM JNJ or 0.1% DMSO (Ct) ERK1/2 activation was monitored at different time points by Western blot. **(B)** Variations in phosphorylated protein levels were quantified by densitometric analysis, normalized with respect to (respective time point) actin and statistically examined by using one-way ANOVA (*p* < 0.001), followed by an SNK *a posteriori* test on log-transformed data. Letters above the bars indicate significant differences (*p* < 0.05). Bars and vertical lines represent the mean ± SEM, respectively (n = 4).

Mitogen-activated protein kinases (MAPKs) play a fundamental role in tumor development (20, 21). Regarding HIS, it was demonstrated in human BC models that blocking H1R induces activation of ERK1/2 and p38 kinases associated with its antineoplastic effect (22). In line with these findings, we found that treatment with JNJ (10 µM) induced a rapid increase in pERK1/2 levels starting at 10 min of 4T1 cell line, which began to decline toward Ct values after 60 min, ultimately returning to baseline levels by 24 h (**Figures 3A, B**). These results suggest that the pro-tumorigenic mechanism of HIS through H4R may involve a negative regulation of these Kinase pathways.

**Figure 4:**
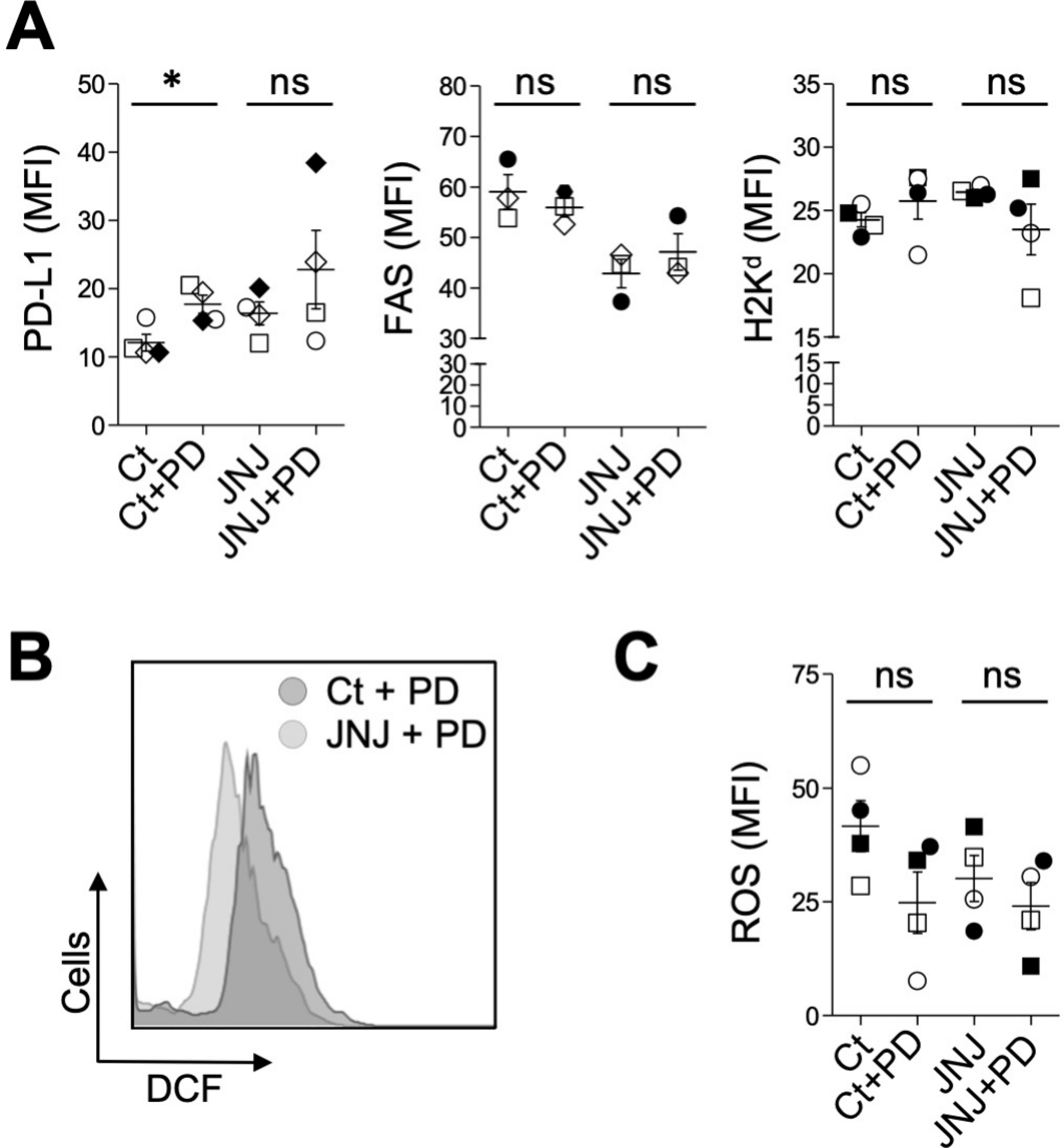
Influence of ERK signaling pathway on JNJ effects. **(A)** Cells were treated with 10 µM JNJ plus PD or 0.1% DMSO (Ct) plus PD for 24 h, and the membrane expression of PD-L1 (n = 4), FAS (n = 4), and H2K^d^ (n = 4) was analyzed by flow cytometry. **(B)** Intracellular ROS generation was evaluated by H_2_DCFDA oxidation after 30 min of treatment with JNJ, with or without PD, and quantified as shown in **(C)** (n = 4). In **(A)** and **(C)**, the effect of PD in Ct and JNJ treatments was determined using Student’s t-test (*p < 0.05). Non-significant differences are indicated as ns. Dots and vertical lines represent the mean fluorescence per cell ± SEM, respectively. Results from the same experiment are represented by identical symbols to illustrate overall consistency across replicates.

To further investigate this point, we used the ERK1/2 phosphorylation inhibitor PD-0325901 (PD). Firstly, we evaluate whether the early and transient JNJ-induced activation of ERK1/2 was related to the expression of membrane proteins. For this purpose, 4T1 cells were pre-incubated with or without PD (2 µM, 20 min at 37°C) and subsequently treated for 24 h with 10 µM JNJ or Ct. As shown in **Figure 4A**, PD effectively reversed the JNJ-induced up-regulation of PDL-1 and class I molecules (*p* < 0.05 and *p* < 0.01, respectively). In contrast, PD did not modify the increase in FAS expression induced by JNJ. Moreover, inhibition of ERK1/2 phosphorylation partially attenuated the JNJ rise in ROS levels (**Figures 4B, C**).

### H4R blockade impairs tumor growth *in vivo* and rapidly promotes CD8^+^ tumor infiltration

**Figure 5:**
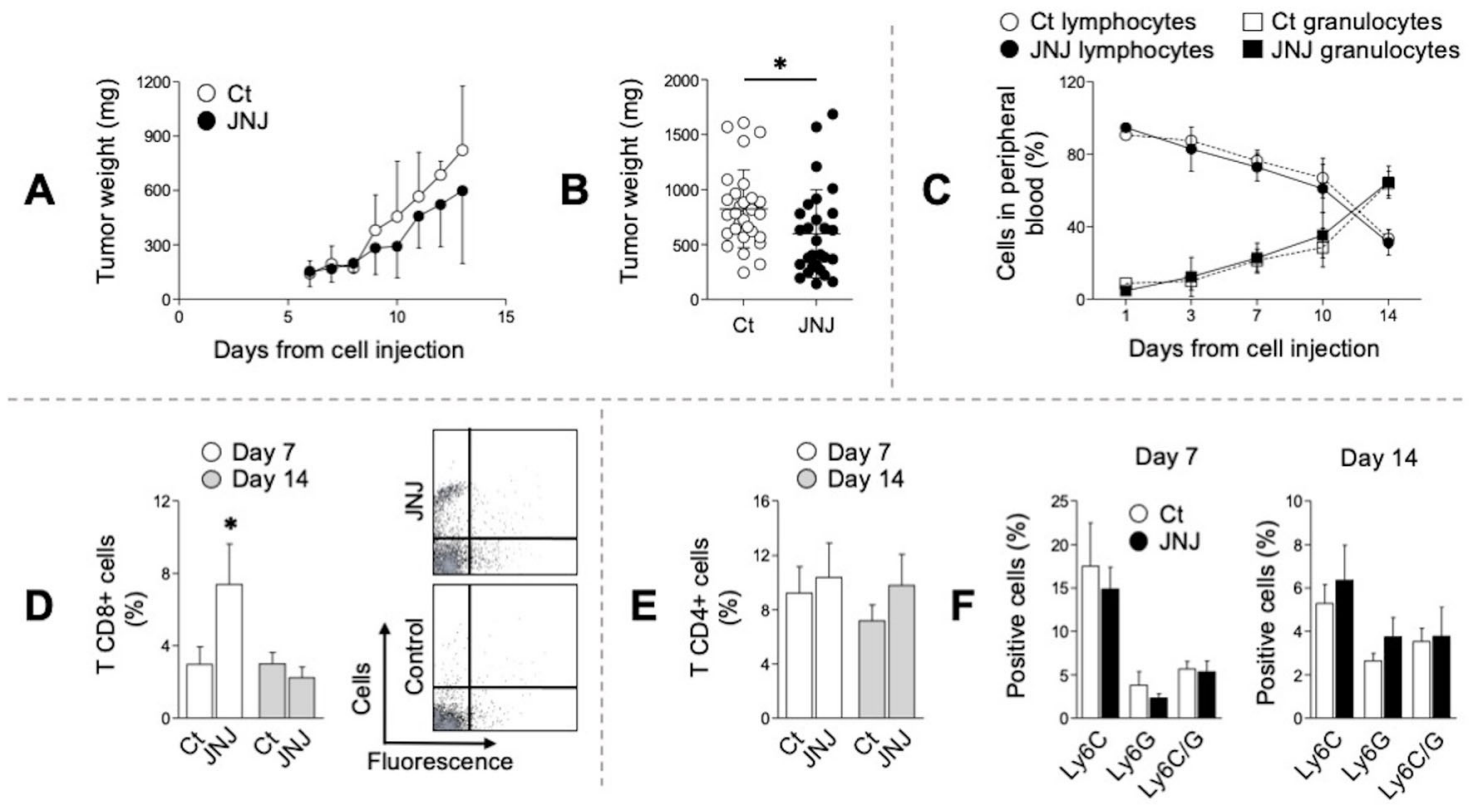
JNJ *in vivo* effect. **(A)** 4T1 cells were inoculated with 10 µM JNJ or vehicle (Ct), and tumor growth was monitored every 2–3 days. **(B)** Variation in tumor mass between experimental groups on day 14 post-inoculation. **(C)** Blood cell counts in animals during tumor development. Recruitment of CD8^+^ **(D)**, CD4^+^ **(E)**, and CD11b^+^/Ly6C^+^/Ly6G^+^ **(F)** cells in neoplastic tissue on days 7 and 14, analyzed by flow cytometry. In **(B, D)** differences between JNJ and Ct conditions were evaluated using Student’s t-test (*p < 0.05).Data are presented as the mean± SEM of 8-10 independent experiments using three mice by treatment, * p< 0.05.

In a next step, we aimed to investigate whether the previous results were transferable *in vivo*. In this context, we employed a syngeneic tumor model by subcutaneously inoculating (10^-5^) 4T1 cells into BALB/c mice with or without JNJ (10 µM) or vehicle (control, Ct). Subsequently, tumor growth was monitored every 2-3 days, along with animal welfare, allowing us to establish the endpoint of the experiment on day 14 after inoculation. At that time, **Figures 5A and B** shows that JNJ induced a significant inhibition (*p* < 0.05) of the 4T1 cells tumorigenic capacity.

The blood count was examined during the aforementioned experimental period. Both treatments exhibited similar pattern of change, and the granulocyte-to-lymphocyte ratio was reversed without significant differences between groups (**Figure 5C**). However, direct analysis of the neoplastic tissue revealed that JNJ induced a rapid and significant (*p* < 0.05) recruitment of CD8 T cells on day 7, which later declined to levels comparable to those of control tumors by day 14 (**Figure 5D**). Conversely, no significant differences were observed between the experimental groups with respect to tumor infiltration by CD4 T cells or myeloid-derived cells (CD11b^+^ Ly6C^+^ Ly6G^+^) either one or two weeks after tumor implantation (**Figure 5E**), nor in the B lymphocyte population or in the production of total IgG (**Supplementary Figure 2A**).

### H4R blockade modulates the BC TME

**Figure 6:**
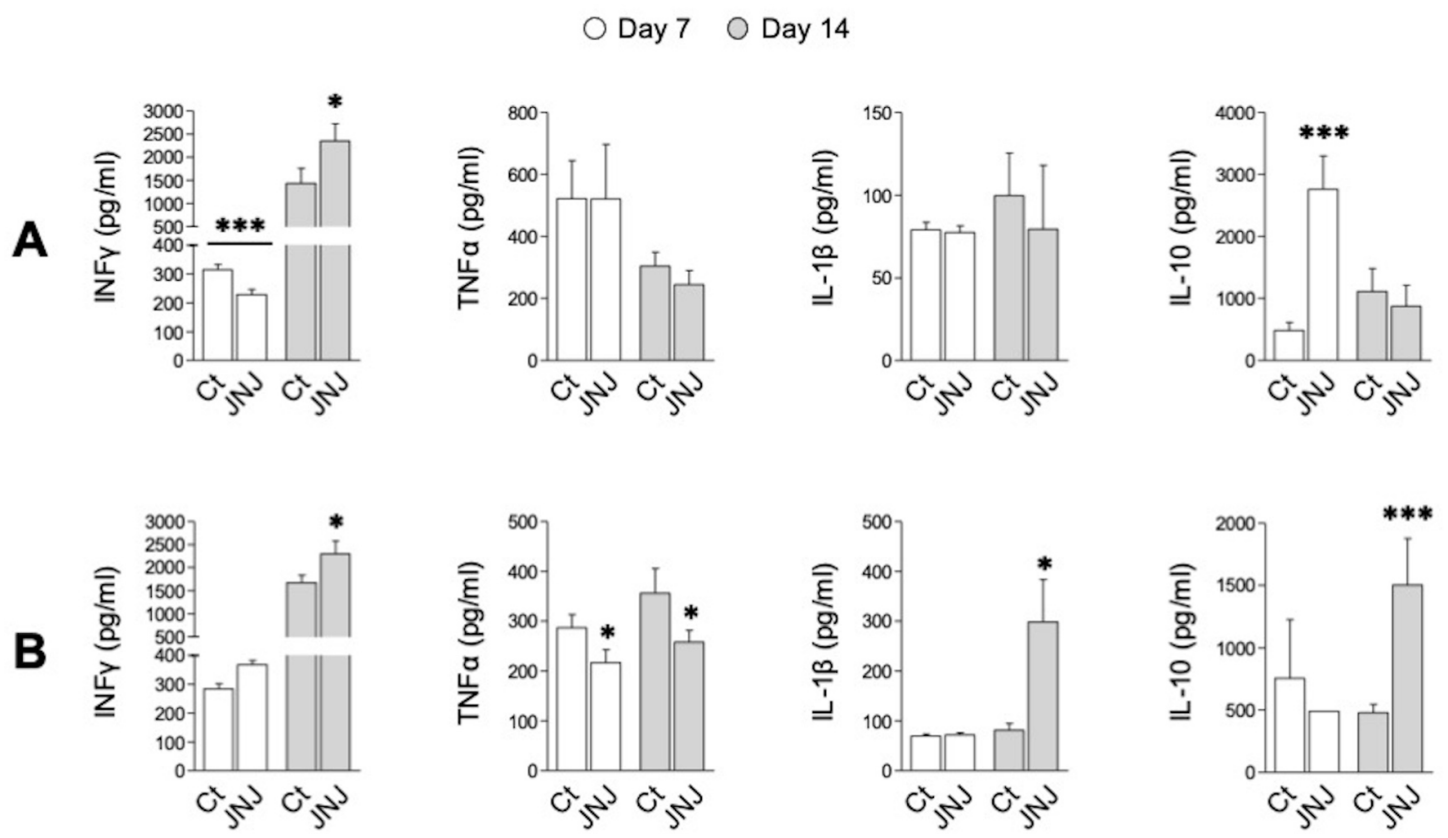
Cytokines secretion in tumor- and lymph node-isolated cells. Samples from control (Ct) and JNJ-treated groups were collected on days 7 and 14 of tumor development. Cells isolated from lymph nodes **(A)** and neoplastic tissue **(B)** were counted and co-cultured with confluent 4T1 cells for 24 h. Culture media were then processed to quantify IFN-γ, TNF-α, IL-1β, and IL-10 protein levels by ELISA. For each time point, statistical differences between Ct and JNJ treatments were determined using Student’s t-test (*p < 0.05, ***p < 0.001). Data represent the mean ± SEM (n = 12).

Cytokine secretion was assessed by ELISA in leukocyte supernatants obtained from tumors and adjacent lymph nodes of mice treated with Ct or JNJ. Mononuclear cells isolated from the tissue were re-exposed to a 4T1 cell monolayer for 24 h. As shown in **Figure 6**, IFN-γ was increased by JNJ (*p* < 0.05) in cells from both tissues on days 7 and 14 (**A** and **B**). Notably, the drug had the opposite effect (*p* < 0.001) in lymph nodes on day 7; this could be due to an early recruitment of lymphocytes to the TME by JNJ.

Supernatants from tumoral cells exposed to JNJ showed a marked reduction (*p* < 0.05) in TNF-α secretion at both experimental time points (**Figure 6B**). However, no changes were observed in lymph node cells (**Figure 6A**). The pro-inflammatory cytokine IL-1β increased significantly (*p* < 0.05) with JNJ in the neoplastic setting during the first week of tumor development (**Figure 6B**). However, its levels were comparable to those of the control group one week later (**Figure 6A**).

Surprisingly, we found that, in addition to IFN-γ, levels of tumor cell-derived IL-10 were markedly increased (*p* < 0.001) by JNJ on day 14. This effect was preceded by a similar rise in IL-10 production in lymph nodes one week earlier (*p* < 0.001) (**Figures 6A, B**).

**Figure 7:**
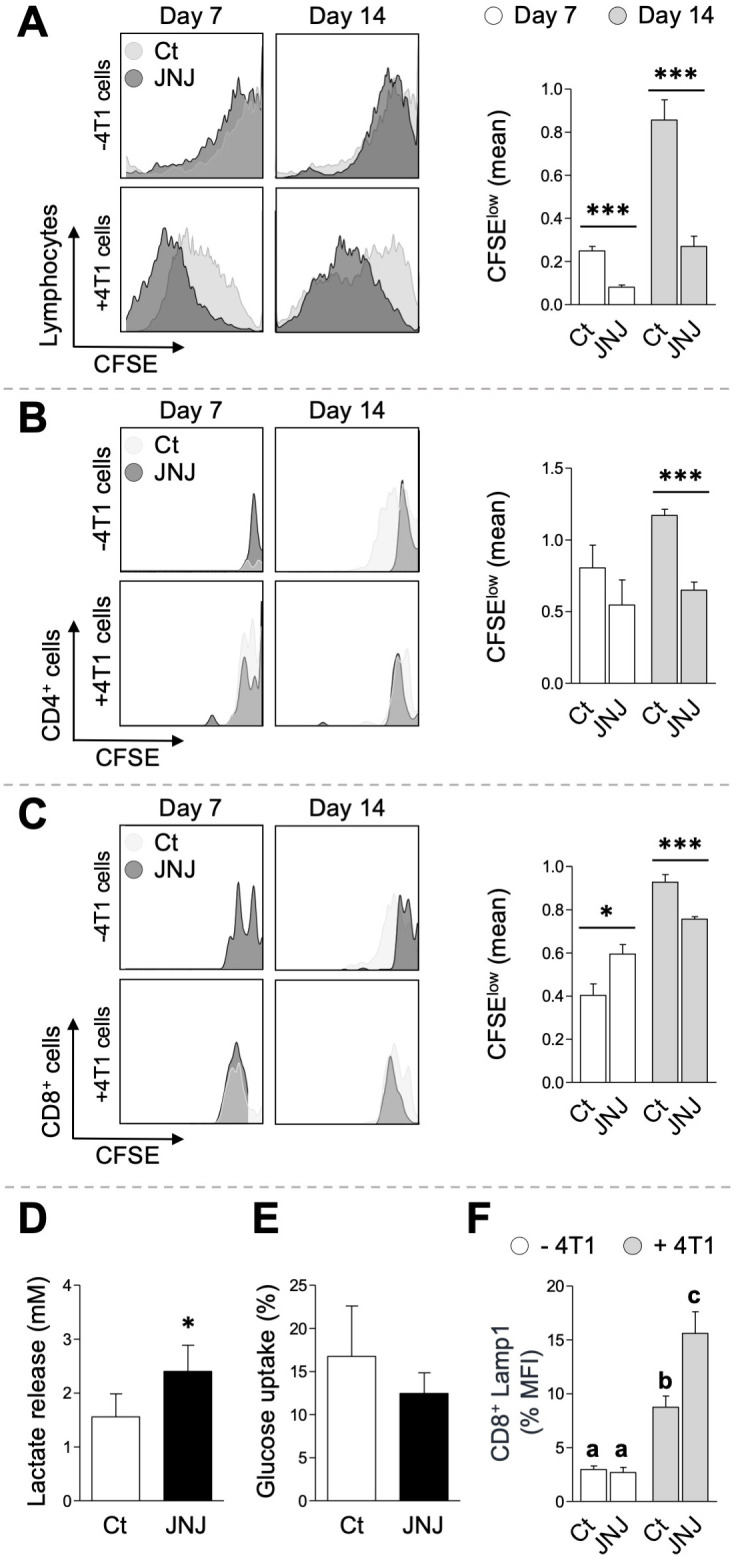
Tumors *ex vivo* studies. Total [**(A)**, n = 20], CD4^+^ [**(B)**, n = 20], and CD8^+^ [**(C)**, n = 20] lymphocytes isolated from tumors at days 7 and 14 (JNJ and Ct groups) were labeled with CFSE and co-cultured with (+) or without (–) 4T1 cells for 96 h. Proliferation ratios, indicated by CFSE dilution through successive cell divisions, were assessed by flow cytometry (histograms on the left). Graphs in **(A–C)** on the right show the variation in mean CFSE fluorescence of lymphocytes from each experimental group, at the indicated times, co-cultured with 4T1 cells relative to their non-stimulated counterparts. Lactate secretion [**(D)**, n = 6] and glucose uptake [**(E)**, n = 6] were measured after a 24-h incubation of tumor-derived lymphocytes. In all cases, differences between treatments were determined using Student’s t-test (*p < 0.05, ***p < 0.001). Bars and vertical lines represent the mean ± SEM, respectively. **(F)** Lamp1 levels in CD8^+^ lymphocytes exposed or not to 4T1 cells for 24 h. Variations between treatments were one-way ANOVA (*p* < 0.01), followed by an SNK *a posteriori* test on log-transformed data. Letters above the bars indicate significant differences (*p* < 0.01). Bars and vertical lines represent the mean ± SEM (n = 6).

In line with these experiments, we evaluated the *ex vivo* proliferative capacity of lymphocytes isolated from tumor tissue. We employed the CFSE staining method, which quantifies the fluorescence dilution resulting from each cell division during 96 h. In this context, lymphocytes obtained from JNJ tumors on day 7 and 14 presented increased proliferation (*p* < 0.001) compared with controls (**Figure 7A**). We next investigated which T cell population was preferentially affected by JNJ treatment. Remarkably, by day 7, JNJ exerted an inhibitory effect (*p* < 0.05) on CD8 T lymphocytes, and that effect was reversed by day 14, when proliferation increased (*p* < 0.001) in both CD4 and CD8 T cells (**Figures 7B, C**, respectively), with the rise being more pronounced in CD4 subset. The early inhibition could be associated with the increase levels of IL-10 detected in cultures supernatants. However, despite IL-10 remaining high in tumor supernatants on day 14, a suppressive T cell response was not induced. In fact, base of these findings, we also evaluated the induction of regulatory subsets. No significant differences were observed in the frequency of FoxP3^+^ CD4^+^ CD25^+^ regulatory T lymphocytes (data not shown).

Similar to what had been previously observed *in vitro*, supernatants from tumors originated by JNJ-treated cells showed increased lactate production (*p* < 0.05), as shown in **Figure 7D and E**. We decided to evaluate the phenotype of effector or resident memory lymphocytes (23) by assessing the early activation marker and the exhaustation receptor, CD69 and Tim3, respectively. As shown in **Supplementary Figure 2**, CD69 expression is not altered in CD8^+^ lymphocytes regardless of the treatment. Regarding Tim3, lymphocytes from JNJ-treated mice have lower expression than controls. Finally, we decided to evaluate the cytolytic capacity of tumor CD8^+^ lymphocytes at 14 days. To do this, we performed a degranulation assay using the CD107a molecule, which is expressed on the CD8^+^ cells membrane upon activation. As shown in **Figure 7F**, CD8^+^ lymphocytes from JNJ-treated mice cultured *in vitro* on a 4T1 cell monolayer for 5 hours exhibited greater lytic capacity than controls, even though the percentage of CD8^+^ cells was similar.

## Discussion

In this study, we decided to evaluate in depth the role of the H4R in BC development in a well-known murine model using the 4T1 cell line. We demonstrated a central modulation of HIS in BC progression associated with proliferation, cell cycle, migration, and immune response through its interaction with the H4R. Several studies have shown that HIS is involved in tumor growth and angiogenesis (24), particularly in BC growth using human and murine cell lines (25, 26). Our *in vitro* assays with the 4T1 cell line showed inhibition of cell proliferation 72 h post-H4 receptor blockade. To our knowledge, this is the first study to evaluate the effect of H4 receptor blockade at the beginning of tumor cell transfer. Interestingly, a single (10 µM) dose of JNJ was sufficient to inhibit tumor growth for at least two weeks. In agreement with our observations, a murine orthotopic model showed that inoculation of 4T1 cells reduced tumor growth in H4R KO mice (11, 12). Conversely, other studies have demonstrated a decrease in tumor growth due to the effect of HIS dihydrochloride (27, 28), a HIS salt used in medicine. It should be noted that the discrepancies between the different groups could be related to the number of cells, the route of drug administration, and the HIS receptor involved. Furthermore, JNJ has been reported to be a partial agonist, depending on the concentrations used, which are generally low or suboptimal (29), which would represent a limitation for the interpretation of our findings. However, it is worth highlighting that, in our model, it acts as a specific antagonist of the H4 receptor since, in proliferation assays, it completely blocks the pro-tumoral effect of HIS (1 µM; data not shown). We also found that the use of JNJ in our murine model was associated with the retention of 4T1 cells in the G0/G1 phase of the cell cycle, indicating a low level of cells in mitosis, which is consistent with the reduced proliferation observed. It is also worth noting that *in vitro* treatment of 4T1 cells with JNJ correlated with lower expression of the cell proliferation marker Ki-67, as assessed by confocal microscopy. (**Supplementary Figure 1**).

In addition to inhibiting tumor growth *in vitro*, as previously demonstrated (24), JNJ could interfere with checkpoints (30–33), facilitating the antitumor effector response. In fact, cancer cells develop mechanisms to evade the immune response, particularly that of T lymphocytes entering the tumor microenvironment. These mechanisms are mainly associated with the modulation of the expression of inhibitory molecules such as PD-1, PD-L1, CTLA-4, or those related to apoptosis, including FAS/FAS-L, among others. Blocking these molecules is precisely the basis of some current anti-tumor immunotherapies. However, not all patients benefit from this treatment, and their efficacy depends on the tumor type malignancy grade (34). In our study, PD-L1 and class I molecules expression in 4T1 cells increased following JNJ treatment *in vitro*. A modest rise in PD-L1 was also detected in tumors from JNJ-treated mice. Nevertheless, given these increases were minimal both *in vitro* and *in vivo*, we do not consider this mechanism to substantially influence the immune response associated with JNJ antagonist.

The MAPK ERK1/2 was associated with proliferation and migration in BC tumor cell lines (35); however, studies analyzing p-ERK levels in patient biopsies (36) yielded contradictory results. We found that the interaction of JNJ with the 4T1 cell line induces transient activation of p-ERK1/2, suggesting a basal role in the negative regulation of HIS via H4R. However, none of the functions evaluated *in vitro* showed a functional association with this pathway. In this regard, although increased expression of ERK1/2 was demonstrated in BC tumor biopsies, this did not correlate with classic ERK-related functions in cancer, such as proliferation (36). Given that HIS signaling via H1R has been shown to involve the activation of ERK and p38 in BC (22), it could be assumed that the classic effects are mediated by the joint activation of both.

CD8^+^ lymphocytes play a fundamental role in antitumor immunity (37). However, many tumors develop mechanisms that prevent effector cells entering the TME from effectively exerting their lytic activity. These mechanisms include the expression of inhibitory molecules, the secretion of anti-inflammatory cytokines, antigenic masking, and the induction of exhausted lymphocytes (37–41). In our model, we observed that pre-inoculation treatment of the 4T1 cell line with JNJ was sufficient to increase early CD8^+^ lymphocyte recruitment. However, the values ​​were comparable to those observed in control mouse tumors two weeks after inoculation. It appears that a longer treatment period is required to maintain the effect over time. Indeed, in most studies evaluating H4 receptor antagonists or H4R-deficient mice, treatments are administered for longer periods in the tumor models studied (5, 11, 12). However, it is worth noting that although the values ​​of tumor infiltrating T lymphocytes in JNJ-treated mice return to levels comparable to those of the control on day 14, these lymphocytes display a greater proliferative capacity when cultured together with 4T1 cells. This finding suggests an increased activation of antigen-specific clones.

Furthermore, after two weeks, we observed an increase in supernatants of IFN-γ, as well as in the anti-inflammatory cytokine IL-10 from leukocytes. Although IL-10 is generally associated with pro-tumor responses (42), Mumm JB et al. (43, 44) recently demonstrated in murine skin tumors that the presence of IL-10 plays a central role in modulating CD8^+^ T lymphocytes, enhancing their cytotoxic activity and promoting IFN-γ secretion.

Besides, we found that tumors from JNJ-treated mice treated increased lactate levels. It has been reported that lactate can favor the anti-tumor CD8^+^ cells response, in part by reducing the acid conditions of solid tumors that would otherwise induce T-cell apoptosis (45, 46). In line with these findings, we observed that although the number of CD8^+^ lymphocytes on day 14 was comparable to that of the control, those from JNJ-treated tumors displayed greater lytic capacity (**Figure 7F**). These data suggest that JNJ could improve the cytolytic response of CD8 lymphocytes, without modifying resident memory (CD8^+^ CD69^+^ Tim3^+^), this could be associated not only with the induction of pro-inflammatory cytokines, but also with metabolic alterations in the TME (46, 47).

A central question in this (*in vivo*) model is whether the antitumor effect of H4R blockade is primarily due to the activation of the immune response, or whether the direct effects of JNJ on malignant cells –such as reduced proliferation and arrest in the G0/G1 phase– are sufficient on their own to limit tumor growth. To address this, we inoculated 4T1 cells, treated or not with JNJ, into RAG1 mice, which lack functional T and B lymphocytes. Tumor growth in these animals was comparable to that of the controls (**Supplementary Figure 3B**), indicating that the adaptive immune response plays a central role in regulating H4R-mediated tumor development.

In conclusion, our findings support a pro-tumoral role for HIS acting through H4R during the early stages of tumorigenesis in 4T1 cells. This effect involves not only a direct action on tumor cells but also modulation of the adaptive immune response, including cytokine production, metabolic reprogramming, and activation of CD8^+^ T cell effector functions.

While H4R blockade may represent a promising antitumor strategy—particularly in tumors characterized by high HIS receptor expression and elevated levels of HIS or its metabolizing enzymes—our results also provide a rationale for, and encourage, further studies using human-derived materials (e.g., cell lines and tumor samples). Such efforts will help to better characterize the influence of the TME, with particular emphasis on the adaptive immune response.

## Data Availability

The original contributions presented in the study are included in the article/**Supplementary Material**. Further inquiries can be directed to the corresponding author.
