# Supplementary material for: Targeting histamine H4 receptor improves anti-tumoral response in a murine model of breast cancer
Source: Front Immunol. 2026 Apr 21;17:1770957. doi: 10.3389/fimmu.2026.1770957 (PMC13139127; doi:10.3389/fimmu.2026.1770957)
Supplement: Supplementary file 1 [file DataSheet1.pdf]

Legends Supplementary Figures

Supplementary Figure 1. Ki-67 and EpCAM expression in JNJ-treated cells. 4T1 cells were treated with 10 µM JNJ or vehicle (Ct), and Ki-67 (**A**) and EpCAM (**B**) levels were evaluated every 24 h by immunostaining. Differences in protein expression were determined by direct cell counting (right graphs in **A** and **B**) followed by Student's t-test (**p < 0.001). Bars and vertical lines represent the mean ± SEM, respectively (n = 3).

Supplementary Figure 2. Lymphocytes phenotype in 4T1 cells tumors. **A**. Percentage of $CD220^{+}$ (B) lymphocytes in tumors on days 7 and 14. **B**. Total serum IgG levels on day 14 post-inoculation. **C** and **E**. A representative fluorescence histogram of Tim-3 and CD69 expression in lymphocytes on day 14 is shown. Statistical differences in fluorescence intensity (mean) between treatments were evaluated using Student's t-test ($p < 0.05$) on log-transformed data. **In D and F,** Bars represent the mean ± SEM (n = 3), *$p < 0.05$.

Supplementary Figure 3. Tumoral growth in immunodeficient mice. **A**. 4T1 cells were treated with 10 µM JNJ or vehicle (Ct) for 30 min, washed, and then inoculated into BALB/c mice. **B**. In parallel, 4T1 cells treated with 10 µM JNJ or vehicle (Ct) were inoculated into Ragl mice. In both cases, tumor growth was monitored every 2–3 days. Variations in tumor mass between experimental groups on day 14 post-inoculation (right graphs in **A** and **B**) were analyzed using Student's *t*-test. Horizontal and vertical lines represent the mean ± SEM (n = 3).

Materials and Methods Supplementary Figure 1

Immunochemistry

Isolated tumor tissue sections were fixed in 4% paraformaldehyde 4 h, followed by incubation in 30% sucrose/PBS overnight at 4°C. finally, tissues were frozen in OCT on dry ice at kept in -80 °C until sectioning. Eight-micron sections were stained with hematoxylin and eosin, Giemsa or fluorescent antibodies. Representative microphotographs were taken with a Ziess Axiolab microscope (Zeiss,Germany). In any cases, Cryosections of 15µm thickness were used to immunofluorescence staining with Epcam, Ki-67 and DAPI. Stained sections were mounted using Aqua-Poly-Mount and microscopically analyzed using FV1000 confocal microscope (Olympus, Tokio, Japan). Sections were later analyzed using the Fiji, Image Sotfware.
