# Supplementary figures and images for: Targeting histamine H4 receptor improves anti-tumoral response in a murine model of breast cancer
Source: Front Immunol. 2026 Apr 21;17:1770957. doi: 10.3389/fimmu.2026.1770957 (PMC13139127; doi:10.3389/fimmu.2026.1770957)

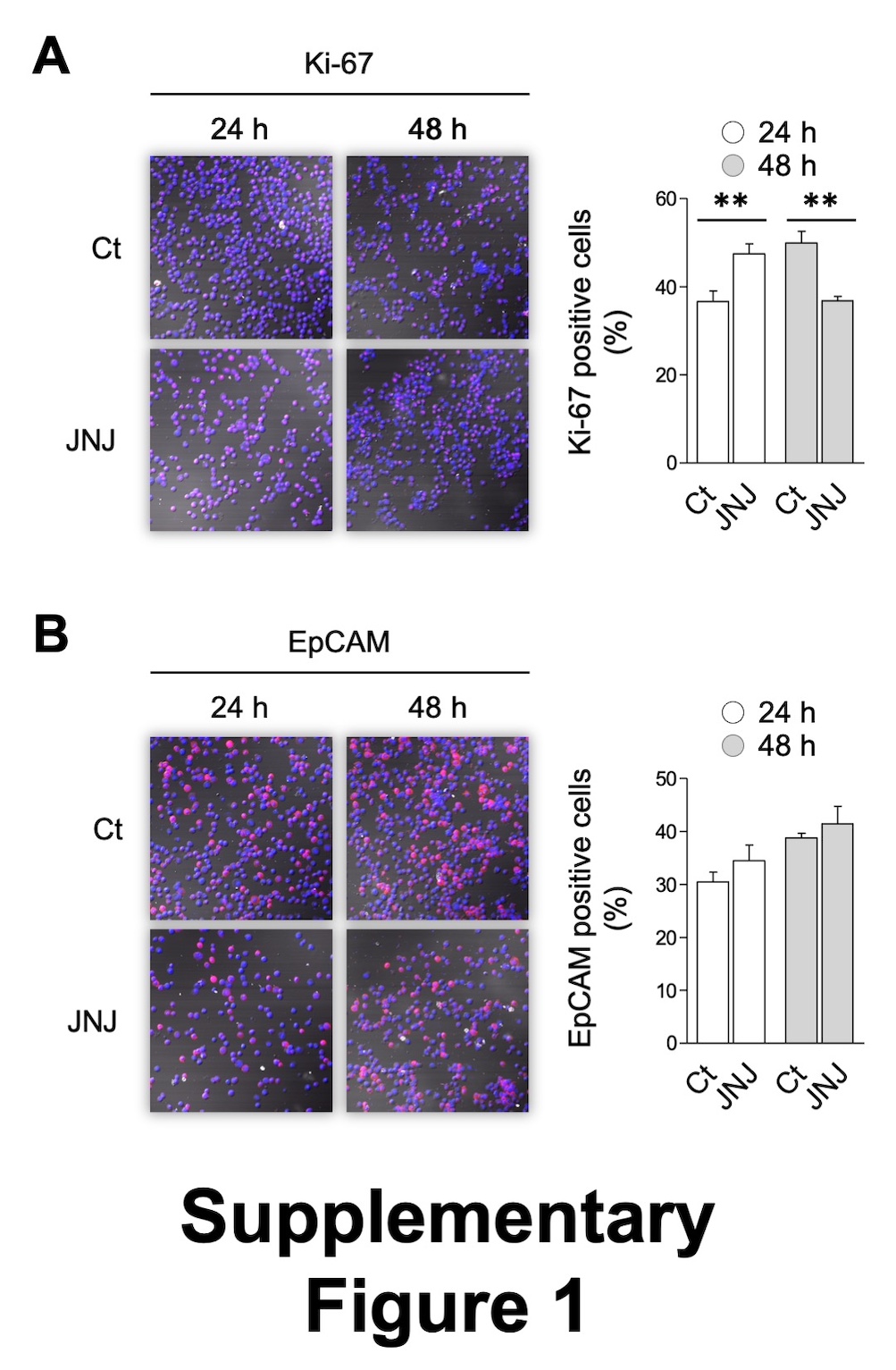

Supplement: Supplementary file 2 [file Image1.jpeg]

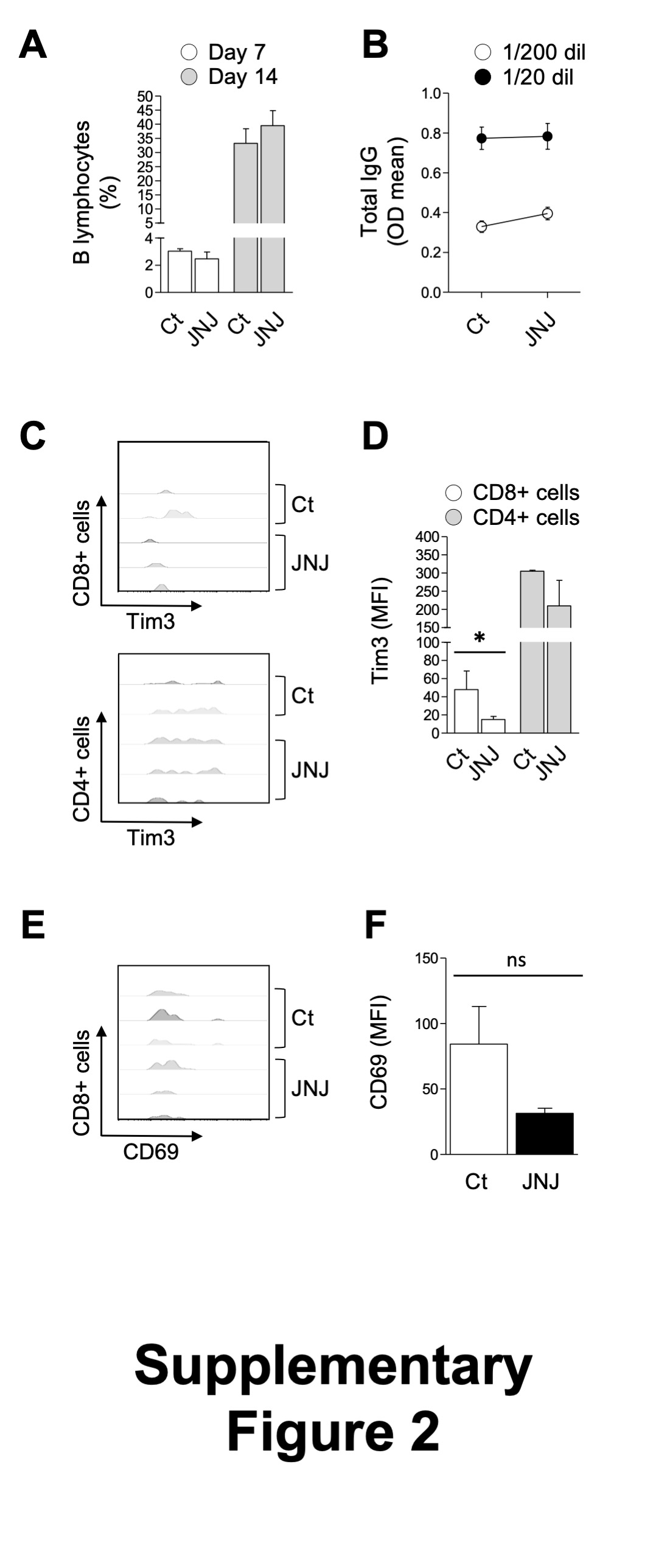

Supplement: Supplementary file 3 [file Image2.jpeg]

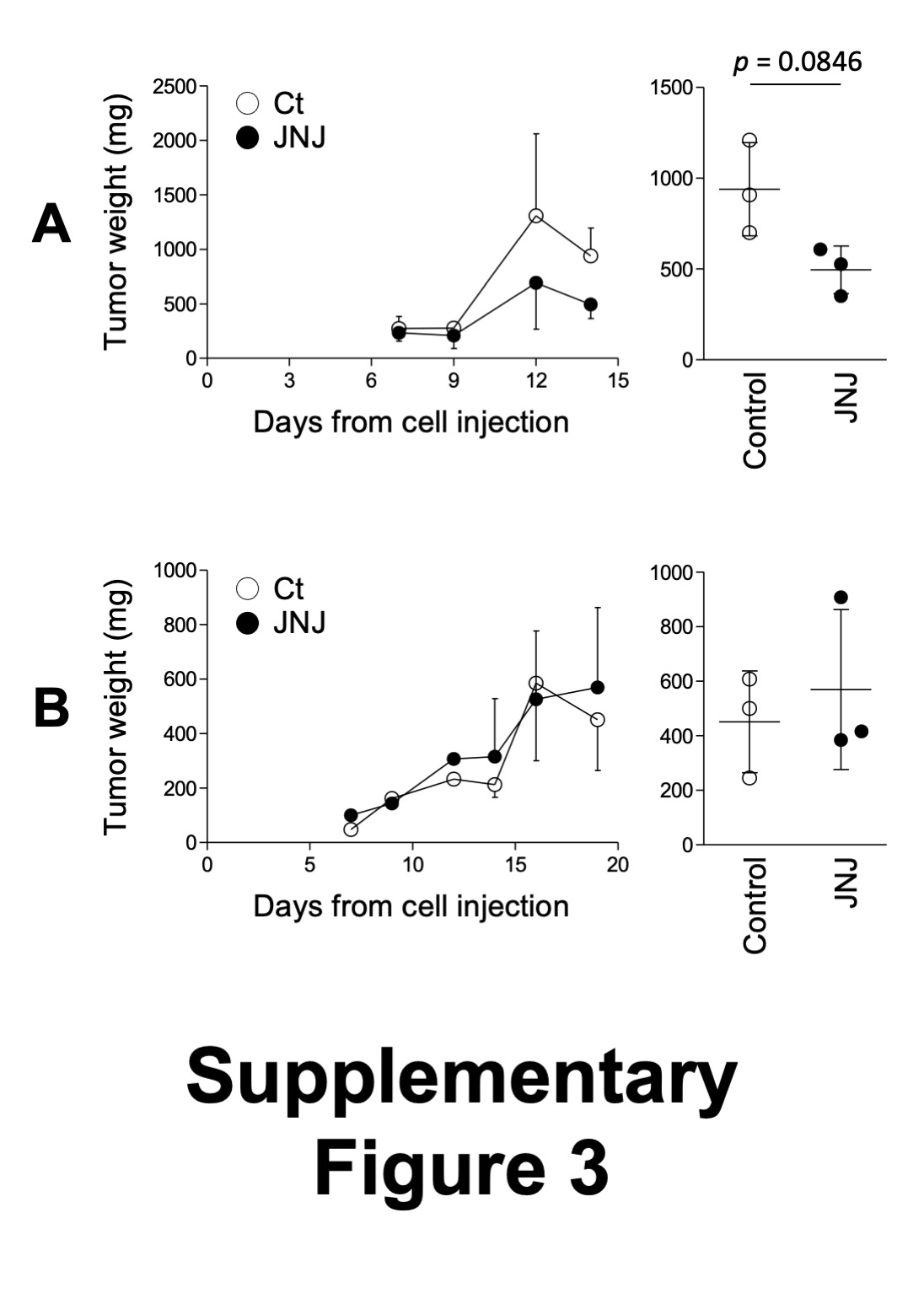

Supplement: Supplementary file 4 [file Image3.jpeg]
