# Supplementary material for: Targeting histamine H4 receptor improves anti-tumoral response in a murine model of breast cancer
Source: Front Immunol. 2026 Apr 21;17:1770957. doi: 10.3389/fimmu.2026.1770957 (PMC13139127; doi:10.3389/fimmu.2026.1770957)
Supplement: Supplementary file 5 [file Table1.docx]

| **Antibody** | **Clone** | **Reference** | **Source** | **Isotype** |
| --- | --- | --- | --- | --- |
| Anti-mouse-FOXP3 | 3G3 | 567462 | BD Pharmingen | Mouse IgG1,κ |
| Anti-mouse-CD11b | M1-70 | 553310 | BD Pharmingen | DA/HA IgG2b,κ |
| Anti-mouse-PD-L1 | 10F-9G2 | 124311 | ebiosciences | Rat IgG2b,κ |
| Anti-mouse-CD8 | H35-17.2 | 550798 | BD Pharmingen | Rat IgG2b,κ |
| Anti-mouse-CD4 | RM4-5 | 561090 | BD Pharmingen | Rat IgG2a,κ |
| Anti-mouse-FAS | Jo-2 | 554258 | BD Pharmingen | Armeniam hamster, IgG2, λ2 |
| Anti-mouse-Ly6C | HK1.4 | B358851 | BioLegend | Rat IgG2c,κ |
| Anti-mouse-Ly6G | 1A8 | B366962 | BioLegend | Rat IgG2a,κ |
| Anti-mouse H2kd | AFB-88.5 | 553457 | BD Pharmingen | RAT (OFA),IgG1,λ |
| Anti-mouse CD25 | PC-61 | 561780 | BD Pharmingen | Rat(Lou)IgG2a,κ |
| Anti-mouse Ki-67 | 16A8 | 652418 | BioLegend | Rat IgG2a,κ |
| anti-mouse  CD107a | ID4B | 553793 | BD Pharmingen | Rat IgG2a,κ |
| anti-mouse CD366 | B8.2C12 | 134011 | BioLegend | Rat IgG1, κ |
| anti-mouse CD69 | H1.2F3 | 104505 | BioLegend | Armenian hamster IgG |
| anti-mouse CD45R/B220 | RA3-6B2 | 553089 | BD Pharmingen | Rat IgG2a,κ |

Table S1: Detail of the antibodies used in the experimental procedure
